# Supplementary figures and images for: Hematopoietic stem cell discovery: unveiling the historical and future perspective of colony-forming units assay
Source: PeerJ. 2025 Jan 29;13:e18854. doi: 10.7717/peerj.18854 (PMC11786707; doi:10.7717/peerj.18854)

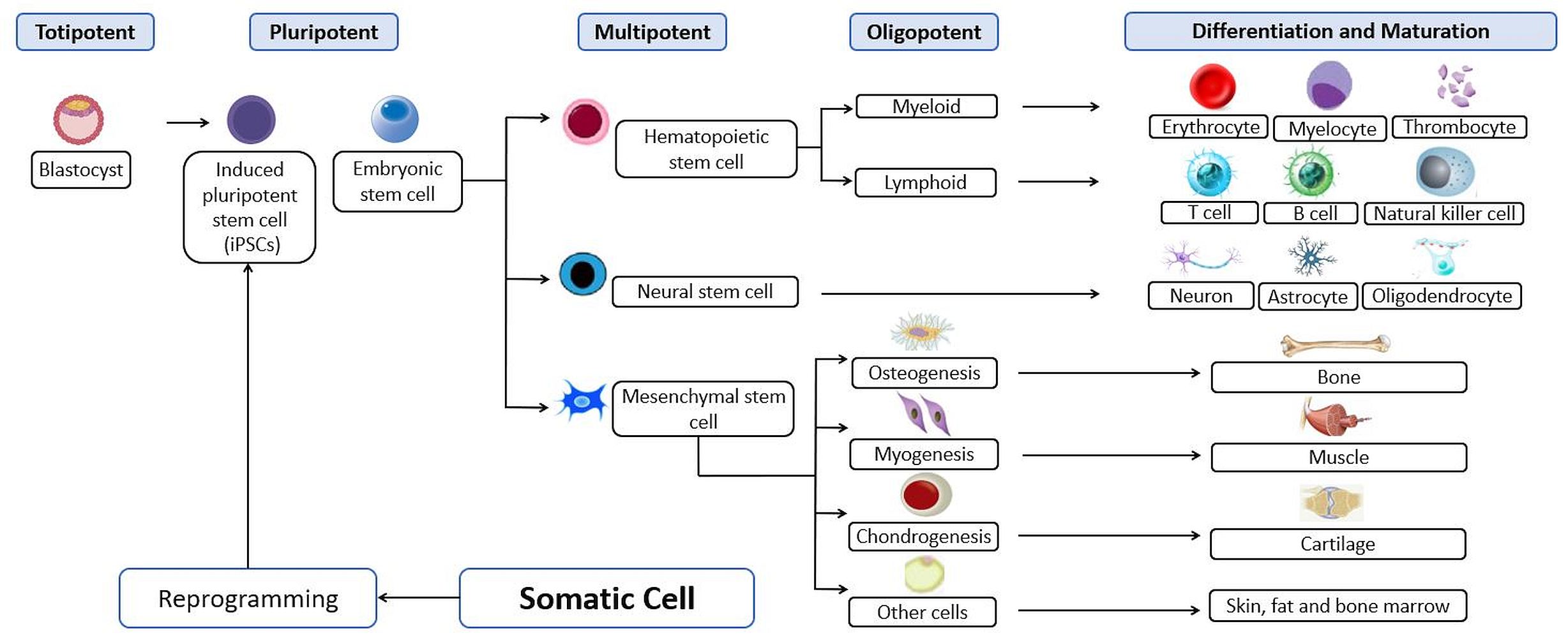

Supplement: Supplemental Information 1 [file peerj-13-18854-s001.jpg]

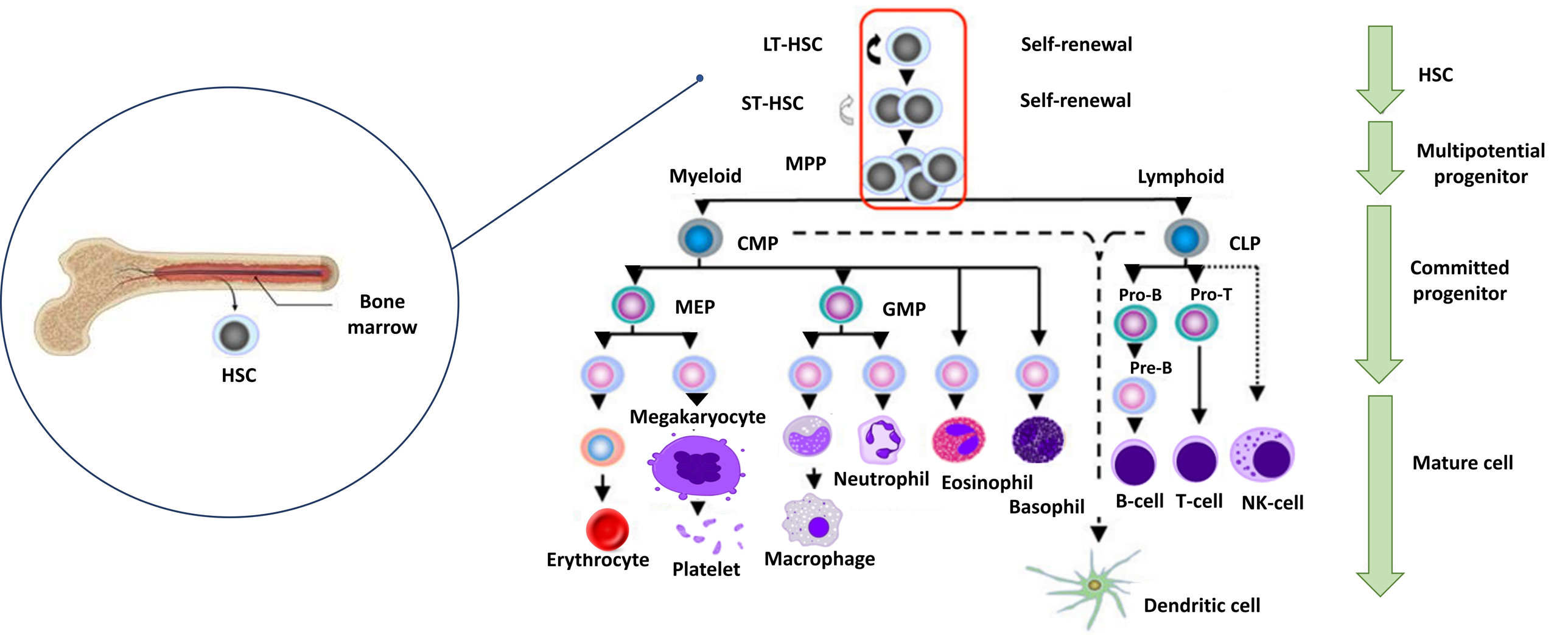

Supplement: Supplemental Information 2 — Modified from (Dewi et al., 2019). [file peerj-13-18854-s002.jpg]

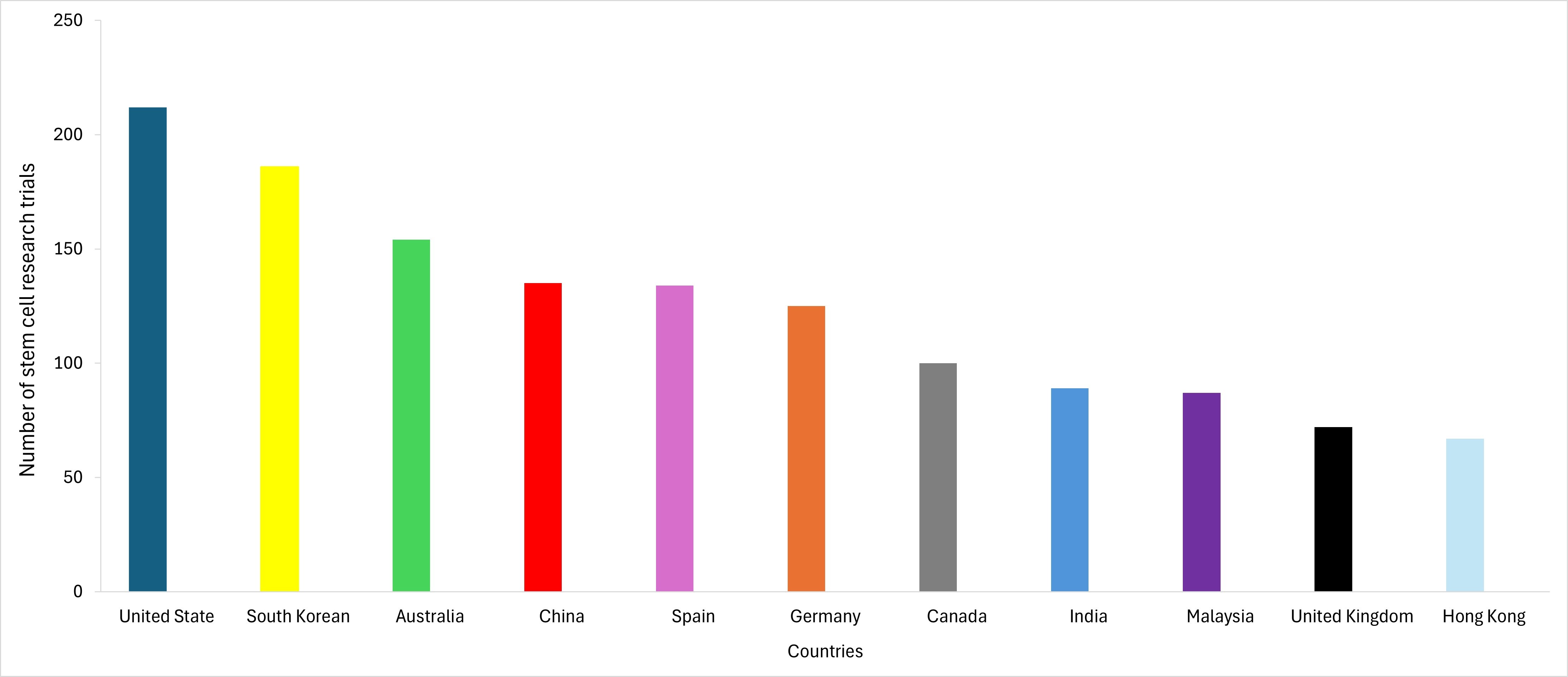

Supplement: Supplemental Information 3 [file peerj-13-18854-s003.jpg]
