# Supplementary material for: Hematopoietic stem cell discovery: unveiling the historical and future perspective of colony-forming units assay
Source: PeerJ. 2025 Jan 29;13:e18854. doi: 10.7717/peerj.18854 (PMC11786707; doi:10.7717/peerj.18854)
Supplement: Supplemental Information 4 [file peerj-13-18854-s004.docx]

**Table S1** **An overview of article selection criteria utilized in this review.**

| **No** | **Category** | **Description** |
| --- | --- | --- |
| 1 | Journal Databases | 1. Google Scholar 2. Scopus 3. PubMed 4. Web of Science |
| 2 | Inclusion Criteria | 1. Articles contain the keywords “colony-forming unit’’, “hematopoietic stem and progenitor cells”, “hematopoietic lineages” and “methylcellulose”. 2. Articles that are linked directly to the utilization and evolution of CFUs assay in analysis of HSPCs along with its role in clinical and basic sciences. 3. The searches were not narrowed down based on specific criteria such as publishing date, authors, author affiliations, journals, or the impact factors of the journals. 4. The quantitative studies that offered measurable data from experimental, epidemiological and clinical research, uncovering observable trends in the use of CFUs assay within stem cell research. 5. The qualitative studies that offered perspectives on the issues and theories that underpin the use of the CFUs assay. |
| 3 | Exclusion Criteria | 1. Articles published in languages other than English, incomplete articles, those with unavailable full texts and unpublished results |
| 4 | Types of articles | 1. 65 original research articles 2. 36 review articles 3. 3 webpages 4. 4 books 5. 2 theses |
