# Supplementary material for: Hematopoietic stem cell discovery: unveiling the historical and future perspective of colony-forming units assay
Source: PeerJ. 2025 Jan 29;13:e18854. doi: 10.7717/peerj.18854 (PMC11786707; doi:10.7717/peerj.18854)
Supplement: Supplemental Information 5 [file peerj-13-18854-s005.docx]

**Table S2 A brief overview of morphological characteristics for respective CFUs.**

| CFUs | Morphology | Characteristics |
| --- | --- | --- |
| 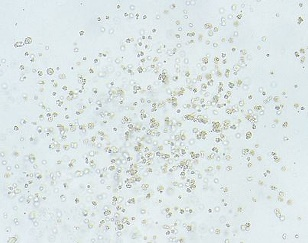  CFU-E |  | CFU-E produces a colony containing 8-200 erythroblasts with 1-2 cells clusters. |
| 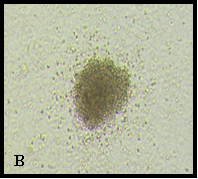  BFU-E |  | BFU-E is more immature erythroid progenitor than CFU-E and produces a colony  containing >200 erythroblasts in a single or multiple clusters. |
| CFU-GM | 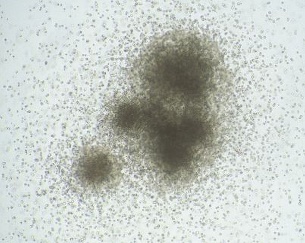 | CFU-GM is more primitive myeloid progenitor than CFU-G and CFU-M and produces a colony with >40 cells of granulocytes and macrophages in a single or multiple clusters. |
| CFU-M | 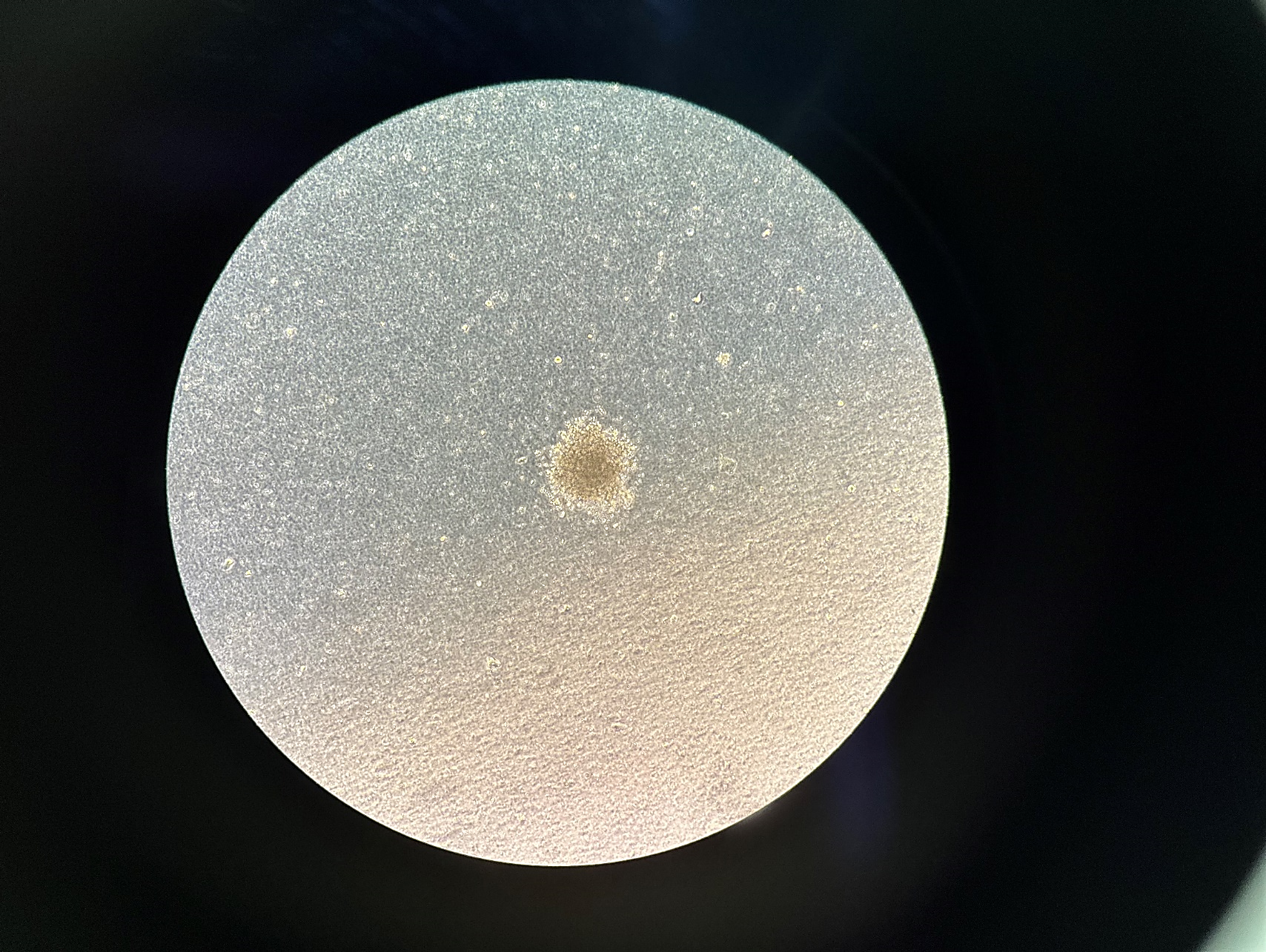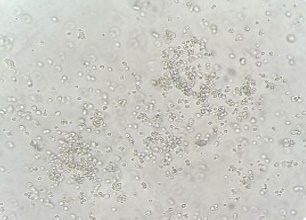 | CFU-M produces a colony containing macrophages with irregular shape and size. |
| CFU-G |  | CFU-G produces a colony containing round and small granulocytes with denser colony as compared to CFU-M. |
| 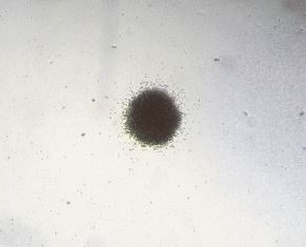  CFU-Pre-B |  | CFU Pre-B produces colonies with variation in size and morphology. Colony consist of tiny, round or oval shaped cells and may appear as compact colonies or diffuse colonies with peripheral cells. |
| CFU-Mk | 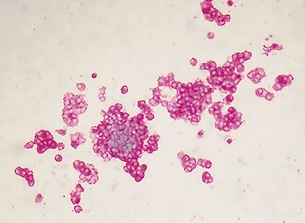 | CFU-Mk produces a colony containing 3 or more megakaryocytic cells. However, the colony requires special staining for megakaryocytes in order to visualize the colony. |
| CFU-GEMM | 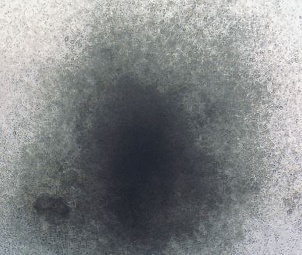 | CFU-GEMM produce large colonies of >500 cells of erythroblasts (at periphery) and lineages (monocytic and granulocytic). Colonies derived from CFU-GEMM have a highly dense core with an indistinct border between the core and peripheral cells. Clusters of large megakaryocytic cells are usually seen. |

**Source:** *StemCell Technologies (2017)*. Abbreviations: CFU-E: colony-forming unit-erythroid; BFU-E: burst-forming unit-erythroid; CFU-GM: colony-forming unit-granulocyte/macrophage; CFU-M: colony-forming unit-macrophage; CFU-G: colony-forming unit- granulocyte; CFU-Pre-B: colony-forming unit-Pre-B; CFU-Mk: colony-forming unit-megakaryocytes; CFU-GEMM: colony-forming unit-granulocyte/erythrocyte/monocyte/megakaryocyte.
